# Ten simple rules for organizing a data science workshop

**DOI:** 10.1371/journal.pcbi.1008226

**Published:** 2020-10-22

**Authors:** Alise Ponsero, Ryan Bartelme, Gustavo de Oliveira Almeida, Alex Bigelow, Reetu Tuteja, Holly Ellingson, Tyson Swetnam, Nirav Merchant, Maliaca Oxnam, Eric Lyons

**Affiliations:** 1 Department of Biosystems Engineering, University of Arizona, Tucson, Arizona, United States of America; 2 School of Information, University of Arizona, Tucson, Arizona, United States of America; 3 Department of Computer Science, University of Arizona, Tucson, Arizona, United States of America; 4 CyVerse, University of Arizona, Tucson, Arizona, United States of America; 5 Data Science Institute, University of Arizona, Tucson, Arizona, United States of America; 6 BIO5 Institute, University of Arizona, Tucson, Arizona, United States of America; 7 School of Plant Sciences, University of Arizona, Tucson, Arizona, United States of America; Dassault Systemes BIOVIA, UNITED STATES

## Introduction

Computational literacy is now a critical skill in most areas of research and commerce [1]. There is a demonstrated need for data science training to bridge domain gaps between physical, biological, and computer sciences [2]. These training sessions can cover a large number of topics such as the use of computational tools in data storage interoperations or the reproducible analysis of large and complex data sets. Short (less than a week long), hands-on workshops offer critical skills development for scientists, at all career stages, outside their work schedules. However, training workshops can be particularly challenging to develop and plan. Often, such events require coordinating a large team of organizers and instructors [3]. Facilitating a data science workshop poses unique challenges due to the field’s large methodological scope. In particular, these trainings need to accommodate participants’ heterogeneous scientific and computational backgrounds, while encompassing the diversity of computational resources and best practices.

Our baseline framework stems from the philosophy and structure used by The Carpentries to teach data and software core skills through hands-on workshops [4]. Their pedagogy utilizes short workshops focused on a specific domain within the contextual framework of the topic being taught (e.g., the complete life cycle of data). The Carpentries curricula for workshops are iterative, and inclusive to people from any background, with no prior experience in the topic area. Their lesson plans are often an excellent place to start finding material when developing a new workshop, though there are many sets of open source materials. Here, we discuss the challenges of organizing participatory data science training and provide 10 simple rules to facilitate inclusive workshops. Each of these rules can be considered independently, but they are written as a progression of ideas that refer back to previously discussed points. Rules 1–6 focus on training material development. Rule 7 is on testing the developed workshop material. Rules 8 and 9 discuss important considerations when running the workshop. Finally, Rule 10 focuses on the importance of continuously evaluating and learning from past workshops to inform future pedagogy.

While short-format workshops are increasingly popular, it should be noted that 1 study showed little difference in long-term performance of graduate students [5]. However, this study notes that longer formats have shown significant positive outcomes, and The Carpentries wrote a thoughtful response [6]. In addition, the authors reference research describing thresholds of learning for graduate students that must be crossed prior to learning specific data analytical skills [7]. While further research into this area is warranted, organizers of data science workshops may consider offering the same material with more spacing between lessons.

## Rule 1: Define the vision and goals

An entire workshop can be challenging to be organized and taught by a single person. The breadth of knowledge required and cognitive biases of a single person could derail a workshop entirely. Therefore, the organization and planning of such workshops necessitate collaboration, as the perspective of different people typically allows to develop a more inclusive training material. Choosing the right people with whom to collaborate is critical, and using campus or organizational networks to connect instructors assures common interests are met. This also facilitates the creation and maintenance of a set of common goals and shared vision across all team members. We believe that the team should develop and document 4 critical areas of the workshop: (1) the vision of the workshop; (2) the goals of the workshop; (3) the expectations from the instructors; and (4) who the target participants are. As outlined in Table 1, we refer to the “goal” of a workshop as the broad concepts that participants should expect to learn from the training. In contrast, the “vision” of the workshop should address why these goals are important; the vision details how the skills acquired will help the participants and meet the values of the training program. Documenting the 4 critical areas will reduce miscommunication between team members, which is critical for workshops lasting more than a few days, or those that are part of a continuous series.

**Table 1:** Definitions and examples of a workshop’s vision, goals, and learning objectives.

Vision	The overall purpose of the workshop. For example, the vision of a git workshop might be to increase participants’ abilities to perform reproducible research.
Goals	The broad concepts that participants should learn that support the vision. For example, a git workshop that introduces participants to version control concepts can increase participants’ ability to understand and track how their data and code have changed over the lifespan of a project—ultimately resulting in more reproducible research.
Learning objectives	The specific skills or operations that participants learn and practice that, collectively, accomplish a goal. For example, in a git workshop, learning objectives might include creating branches, staging changes, merging branches, handling merge conflicts, and pushing to or pulling from remote repositories. Together, these learning objectives accomplish the goal of introducing participants to version control concepts.
Blameless retrospective	Blameless retrospectives originate from Agile-based software development. Their goal is to create a group culture of psychological safety and accountability and facilitate continuous learning. In these retrospectives, it is often useful to refer back to the project management plan and determine which strategies facilitated learning objectives, goals, and contributed to the workshop’s vision.

## Rule 2: Divide the workload into teams and use team-based approaches to collaborate

It is critical to acknowledge the time and effort required to prepare, test, and teach the workshop. These events are a great opportunity for students and researchers to develop their teaching and communication skills. Importantly, newcomers to instruction should be welcomed and mentored. New instructors may want to review the instructor training material developed by The Carpentries [8] to get familiar with inclusive teaching methods. We recommend that experienced instructors focus on setting the overall tone for the workshop, while providing opportunities for less experienced people to assist in teaching materials.

In order to alleviate the workload associated with developing the workshop’s training material, we recommend a modular approach for developing the teaching material. In this framework, each training module is created and taught by independent teams. The team should refer back to the common guideline developed for the overall workshop (Rule 1) while defining each module’s scope and goals.

We believe that smaller teams and modularity of lessons facilitate efficient project management practices. It is important for the instructors to establish a schedule for the development of training modules as well as provide time for testing new materials. Organizational management tools like Zenhub (https://www.zenhub.com), Trello (https://trello.com), or Basecamp (https://basecamp.com/) can help the coordinator track progress while the workshop is in development. As workshop development matures, these tools can also provide a place to develop backup plans for alternative locations, equipment, instructors, etc. This is also important when teaching online workshops, which can face their own specific technical hardships. Ultimately, good project management tools ensure overall workshop leadership can track all teams’ curricula developments, while also creating a unified communication framework and an explicit schedule of the workshop events.

## Rule 3: Ensure sessions are connected

We recognize a potential pitfall of developing modular training material (Rule 2) could be differences in presentation style, supporting documentation, and learning objectives. Connecting learning objectives from different training modules taught by different instructors requires a large effort in coordination and communication. Therefore, a person should be designated as the overall coordinator to ensure learning objectives from individual training modules are connected. For smaller workshops, this coordinator may also work in an instructor team. The coordinator’s work can be facilitated through project management tools outlined in Rule 2, and ideally, the coordinator would act as the project manager. Finally, the workshop coordinator should ensure each session and learning objectives build toward the overall vision and goals of the workshop (Rule 1).

## Rule 4: Communicate the learning objectives

The workshop coordinator ensures the connection of the different modules developed by the team (Rule 3). However, these connections should also be evident to the learner—it can be easy for both instructors and participants to lose sight of learning objectives, leading to many other pitfalls. An overview of skills in each session helps ground participants’ expectations and can serve as a reminder to instructors of the most important points that they need to cover. The interrelatedness of the modules covered should be referenced as each session closes, which should be connected back to the vision and goals of the workshop (Rule1).

The learning objectives can be defined as the specific skills that participants will learn during the workshop. In a git workshop, the learning objectives might include creating branches as well as pushing to or pulling from remote repositories. The workshop’s main learning objectives could be presented as an overview at the workshop’s opening, along with introducing the instructors, the overall schedule, and a discussion on the code of conduct. However, the learning objectives should also be emphasized during and after each module and recapitulated during the workshop’s closing remarks. At the beginning of each training module, instructors should clearly describe learning objectives and the skills acquired during this session (Table 1). A record of learning objectives should be provided in a document specifying low-level objectives (e.g., learning the commands “ls” and “cd”) as well as high-level workshop goals (e.g., learn to use commands together for automation of computational tasks). Sharing these documents ensures all instructors are made aware of other sessions’ objectives, avoiding overlap or gaps in knowledge across sessions.

## Rule 5: Implement active learning sessions

Data science workshops will have a blend of conceptual overviews (e.g., why a particular set of technologies is used and how they fit together), best practices (e.g., why containerization is useful for reproducible computing), and technical hands-on exercises (e.g., pulling docker containers and creating new dockerfiles). Across the developed training modules (Rule 2), active learning helps participants understand the rationale behind learning objectives and technical choices of the module (Rule 4). Felder and Brent (2009) define active learning as anything course related that all students in a class session are called upon to do rather than simply watching, listening, or taking notes [9]. In addition, the instruction should provide learners not only with practice implementing technologies but also how to find help autonomously to resolve technical issues after the workshop adjourns.

The active learning environment is enhanced by developing group or live coding problem-solving activities in a physical environment that facilitates cooperation (e.g., tables, computer displays and whiteboards, and breakout areas) [10]. In online workshops, these activities can be done by creating small subgroups of participants working together or with individuals working simultaneously on the same problem set. It is essential to recruit helpers/facilitators familiar with the technologies to ensure adequate workshop pacing and assist with unforeseen technical problems [11]. Additionally, it is important to allow for helpers and facilitators to easily move between individuals/groups and track their progress, which can be particularly challenging to monitor in an online setting.

Instructors may underestimate the time required for an active learning module. This is especially challenging when instructing in a heterogeneous computing environment with multiple software code stacks. Instructors should be mindful of the pace and watch for participants falling behind the rest of the group. It is important to ensure participants feel comfortable in speaking out with their difficulties, which can be onerous in some online settings. It can be beneficial to encourage participants to keep their cameras on to ensure engagement and allow a better gauge of their status. This may be impossible with larger groups or with slow internet connections. Additionally, we recommend providing instructions before the workshop on how to use interactive features of online video conference tools (e.g., attendees raise hands, polling, and breakout rooms).

## Rule 6: Evaluate and teach to participants’ skill sets and expectations

Workshop participants are likely to come from diverse scientific backgrounds with varying levels of computational literacy. It is critical to understand their expectations and evaluate their skill sets to ensure that the workshop material and active learning sessions meet the needs of participants [3]. In addition, instructors must have empathy for participants and consider the learner’s expectations when conducting their presentations. When organizing virtual workshops, keep in mind that participants may have limited resources that can hinder their learning experience (such as having a small, single monitor, slow computer, or limited bandwidth). In addition, it is important to make sure that the workshop is accessible for all participants [12,13]. We recommend providing a pre-workshop survey to participants using the workshop goals (Rule 1) to shape the questions. If the workshop is part of a series, questions like “Are you participating in the next workshop on topic X that builds from this content?” can better link workshop contents over disparate time scales. Interested readers can find here an example of survey here: http://bit.ly/DS-Qs. Collecting participant’s feedback allows instructors to better develop active learning modules matching the participants’ domains of interest (Rule 5) and provide information for refining learning objectives (Rule 4).

## Rule 7: Test workshop materials and installation with a cognitive walkthrough

Prior to your workshop, it is important to test new teaching materials with naïve users (defined as anyone with no prior experience) in the specific domain, software, or technique being taught. The material should be complete and be easily followed by someone who is new to the field. Particular care should be taken during this test to ensure the lesson will fit in the allotted time. There should be no gaps in knowledge and/or lesson steps. Review the connections of learning objectives for each module to the needs identified in a pre-workshop survey (Rule 6). While pretesting the learning material is critical for the workshop's success, it is also extremely time consuming. We therefore advise all instructors to deliver their material at least 2 weeks prior to the workshop to ensure sufficient time for cognitive walkthroughs.

The particularities of each operating system should be taken into account when the participant is expected to provide their own laptop. In particular, installation procedures should be detailed for each platform. If the learner is expected to complete the computational tool installation prior to the workshop, plan at least an hour or 2 on the first day to ensure that all required software and dependencies are configured. If the workshop provides computers, a system support technologist should be available at the beginning of the workshop to help with any system issues [3].

It is important to note that many data science tools and platforms are complicated stacks of software, which can be difficult to install from scratch. It may be important to preinstall these on computers used in the workshop or provide preconfigured virtual machines and/or containers (Docker (https://www.docker.com/)/Singularity (https://singularity.lbl.gov/)). The chosen toolset informs skill prerequisites for participants prior to a module being taught (e.g., linux command line, logging into remote servers, etc.). These skills will need to either be covered early in the workshop or be expected as workshop prerequisites.

## Rule 8: Stick to the script

Be mindful that workshop material should be understandable for all learners (their prior knowledge should be informed by Rule 6). Presentation slides should include the most important points, since oral comprehension can be augmented with written notes. It is important for instructors to speak slowly, clearly, and avoid “going off script.” We define “going off script” to be the inclusion of technical material outside the scope defined by the workshop (Rules 2–5). If instructors include steps not clearly documented in the supporting material, this could lead to confusion, take extra time, and be particularly difficult for nonnative language speakers. Such areas can be identified before the workshop during cognitive walkthroughs (Rule 7). All instructors must understand that adding any additional information outside the written material and learning objectives of a module may be of little value to participants. Participant questions may lead to an instructor “going off script,” and while these discussions may be useful, the instructor should reserve the right to address their question during a dedicated time for questions and answers or one-on-one during a break. However, this is not meant to impose any restrictions on personal style of presentation. We strongly encourage presenters to find their own voice and not simply read bullet points on a slide. Dynamic and interactive presentation is a skill that the instructors are practicing and honing while teaching these sorts of workshops.

## Rule 9: Select and share external learning resources opportunities

In addition to developing their own teaching material, the instructors should strive to reference external resources available to participants. These workshops often provide foundational skills, but adding links to external resources will empower participants to continue their learning according to their needs and interests [3]. This also allows engaged or more advanced participants to access content beyond the scope of the workshop. In addition, these resources can be referenced if questions cause the module to go off script (Rule 8). The pre-workshop survey (Rule 6) ensures extra content is tailored to extend the participants’ learning trajectory beyond the workshop. When all rules of workshop organization are met by the organizers, the participants will leave with a sense of starting a journey toward a new long-term goal for themselves or their research group.

## Rule 10: Take into account evaluations from previous years and hold a blameless retrospective analysis soon after a workshop

Workshops are rarely developed perfectly from the onset; learning from past experiences is critical to improving workshop content. As such, it is important to have surveys for each module and the workshop as a whole [14]. We recommend reserving time for these to be completed during the workshop. For example, if holding a multiday workshop, set aside 10 minutes in the morning of each day to let participants review the previous day’s modules. These day-by-day evaluations facilitate tracking potential issues and implementing possible course corrections. In addition, it is important to hold a blameless retrospective (Table 1) [11,15]. There are several ways to conduct a blameless retrospective, but generally, they should be critical, relaxed, and provide a forum to identify what worked well, what didn’t work well, and what should be done differently the next time the workshop is taught. When the retrospective concludes, there should be a list of action items to help with the planning and execution of future workshops. These assessments should aim to inform the practice of the next workshop and provide guidance for the instructors, allowing them to revise their style and approach in the future, and therefore should be documented and made accessible for future instructors. This practice, and revisiting Rules 1–9 at this phase, will ensure that organizers, instructors, and helpers continue to refine, rather than reinvent, prior workshops. In particular, workshop organizers should pay attention to differences in learning objectives between iterations of workshops. This practice allows instructors to not be as constrained by prior editions of the workshop, since computational tools and data science are rapidly changing fields.

## A note: An increasingly virtual world

This paper was developed and written before the Coronavirus Disease 2019 (COVID-19) pandemic, which has forced many workshops and training into a virtual environment. We believe that all of the rules presented here are still useful in guiding the organization and implementation of a workshop in a virtual environment but do not cover all the unique challenges remote training face. The presented rules aim to help create a well-organized workshop with clear intentions and an empathetic environment in which participants feel both productive and understood. This focus on accessibility is particularly important to reduce the personal distance inherent in online workshops. Additionally, these recommendations should guide organizers to provide complete and accessible materials for reference, a point particularly important for remote teaching, where home life can frequently interrupt participation. Although not discussed here, it is also worth noting that instructors of online workshops should design their teaching with an extra effort on interactivity and checking for participants’ understanding frequently. There are many resources available to help increase interactivity of online workshops, and we encourage the reader to try and choose the platform and tools that best meet their needs. We cannot wait to read the 10 simple rules papers summarizing what the community will learn about remote teaching during these exceptional times.
